# Primer evaluation and development of a droplet digital PCR protocol targeting *amoA* genes for the quantification of Comammox in lakes

**DOI:** 10.1038/s41598-021-82613-6

**Published:** 2021-02-03

**Authors:** Manuel Harringer, Albin Alfreider

**Affiliations:** Department of Ecology, University of Innsbruck, Innsbruck, Austria

**Keywords:** Ecology, Microbiology, Ecology, Limnology

## Abstract

To date, little is known about the ecological significance of Comammox (COMplete AMMonia OXidizers) *Nitrospira* in the water column of freshwater lakes. Water samples collected along depth profiles were used to investigate the distribution of Comammox in 13 lakes characterized by a wide range of physicochemical properties. Several published primers, which target the α-subunit of the ammonia monooxygenase, generated non-specific PCR products or did not amplify target genes from lake water and other habitats. Therefore, a new primer set has been designed for specific detection of Comammox in lakes. The high specificity of the PCR assay was confirmed by sequencing analysis. Quantification of Comammox *amoA* genes in lake water samples based on droplet digital PCR (ddPCR) revealed very low abundances (not exceeding 85 *amoA* copies ml^−1^), which suggest that Comammox is of minor importance for the nitrification process in the water column of the study sites. Surprisingly, samples taken from the sediment/water-interface along an oxygen gradient in dimictic Piburger See showed Comammox abundances three to four magnitudes higher than in the pelagic realm of the lake, which indicates a preference of Comammox to a particle-attached lifestyle.

## Introduction

Nitrification, the oxidation of ammonia to nitrate via nitrite, is a key process of the global nitrogen cycle in aquatic and terrestrial ecosystems^1–3^. Since the discovery of nitrification more than 100 years ago^4^, both steps of nitrification have been considered to be catalysed by different chemolithoautotrophic microorganisms: ammonia-oxidizing prokaryotes (AOP) including Archaea (AOA) and bacteria (AOB) catalyse the first step, which leads to the formation of nitrite. In the second step of nitrification, nitrite is further oxidized to nitrate by nitrite-oxidizing bacteria (NOB)^5,6^. The recent discovery of microorganisms that can perform both steps of nitrification (complete ammonia oxidizers; Comammox) challenged this dogma and has significantly changed our perspective on the nitrification process^7,8^. To date, all Comammox described so far belong to the genus *Nitrospira*^7,8^, one of the most diverse taxa among NOB^9,10^. Since the description and isolation of the three *Candidatus* species obtained from an oil exploration pipeline biofilm^7^ and a trickling filter from an aquaculture system^8^, research of the Comammox process is currently heavily biased towards engineered systems. Particularly, waste water treatment plants are often used as model systems to study niche preferences and environmental adaptations of Comammox *Nitrospira*^8,11–16^. In natural environments, most studies are focusing on soil habitats^8,11,17,18^, whereas fewer investigations have been conducted in aquatic ecosystems^11,18–22^.

Lakes often develop stratification patterns during the summer season, which results in the formation of pronounced physical and chemical gradients inhabited by nitrifying microorganism^23^. Generally, AOB are the dominant group of AOP at conditions with high ammonia concentrations^24^ in smaller lakes, whereas AOA are the major group of AOP in the oxygenated hypolimnion of deep oligotrophic lakes^25–27^. Studies that investigate the occurrence of Comammox in lakes are rare, so far first results provide no clear picture on the distribution of Comammox in the water column of lakes^18,26,27^. Only one study^18^ identified high numbers of Comammox (up to 10^6^ *Nitrospira amoA* copies l^−1^) in lake water samples, in contrast to most other investigations where Comammox were absent or barely present in the pelagic zone of lakes^26,27^.

For the detection and quantification of Comammox, different marker genes have been tested. Traditional taxonomic markers targeting genes that encode the 16S rRNA do not allow the differentiation between Comammox and canonical *Nitrospira*-NOB, because Comammox do not form a monophyletic clade within *Nitrospira*^7,8,11^. It was also reported that phylogenies based on sequences of the ATP citrate lyase and nitrite oxidoreductase, which are essential enzymes of the reductive tricarboxylic acid cycle (rTCA) to assimilate inorganic carbon and the nitrite oxidation, do not allow to distinguish between Comammox and nitrite-oxidizing *Nitrospira*^7,8,11^. Genes that encode for subunits of the ammonia monooxygenase (AMO), and especially those of the α-subunit (*amoA*), are widely used for the detection and quantification of aerobic ammonia oxidizers^28–31^. Comammox harbour novel forms of AMO, therefore analysis based on *amoA* sequences enable to differentiate between Comammox and canonical AOP^7,8,11^. At present, several Comammox specific primer sets have been published, including highly degenerated primers targeting one or both clades of Comammox^11,15^ and species-specific primer sets^12,16^. However, it has been reported that degenerated primers resulted in significant non-specific amplification when used for the detection of Comammox in samples from activated sludge^16^. This could be explained by the high sequence similarity between genes coding for *amoA* and the α-subunit of the particulate methane monooxygenase (*pmoA*), increasing the risk of false-positive amplification with increasing primer degeneracy^32^.

Here, we report the results of an investigation that aims to quantify Comammox in the water column of 13 stratified lakes. One lake (Piburger See) was selected to obtain additional information on the spatiotemporal distribution of Comammox and to address their potential occurrence along a gradient in the sediment/water-interface. Already published Comammox *amoA* primer sets were tested regarding their ability for the specific detection and quantification of Comammox in lake water samples but also in samples from other habitats. Furthermore, a highly specific new primer set was designed, based on *amoA* sequences derived from lake water samples. These primers enabled us to quantify Comammox *amoA* gene copy numbers by droplet digital PCR (ddPCR) in samples obtained from lakes with different physicochemical properties, in order to get information on the distribution and potential niche preferences of Comammox in lakes.

## Results and discussion

### Physicochemical lake characteristics

The physical and chemical properties of the lakes sampled once during summer stagnation and the pelagic water samples obtained over an annual cycle from PIB (lake abbreviations see methods section) were described in detail in former studies^25,26,33^. Briefly, large and deep lakes HAL, MIL, MON, TRA and ZUR showed a significant decline of dissolved oxygen (DO) with depth, while ACH and ATT were well oxygenated over almost the entire water column. The hypolimnion of WEI and the smaller lakes EGE, FAA, HEC, IRR, was characterized by pronounced differences in the extent of the anoxic zone. Correspondingly, most lakes showed a significant increase in ammonium concentrations with depth. The annual stratification pattern of PIB is characterized by an inverse temperature stratification during winter under the ice cover, while summer stratification was observed from April to early November. During summer, DO in the hypolimnion was strongly depleted and the anoxic zone reached up to 18 m depth, which was associated with an accumulation of ammonium. The samples of the sediment/water-interface (SWI) showed a clear decrease in the DO concentration with increasing depth, covering a range from 0 to 10.42 DO mg l^−1^ (Fig. 4).

### In vitro evaluation of the specifity of existing primers for the detection of Comammox in environmental samples

The highly degenerated primer pair published by Fowler et al*.*^15^ (Ntsp-amoA 162F/359R, Table 1) was designed for simultaneous detection of clade A and B of Comammox. The primer pair was tested with two samples of PIB (June and July at 21 m depth) and five non-lake environmental samples. The agarose gel performed with the PCR products obtained from a variety of samples revealed DNA-bands of different lengths, with exception of the biofilter (BF) sample (Supplementary Fig. S1). The non-specific amplification with this primer set was already described in a previous study^16^. Sequence analysis of amplification products showed that less than half (26) of 59 clone sequences had a correct fragment length, while the remaining sequences represented unspecific amplification products. The phylogenetic reconstruction of sequences with proper length showed that sequences obtained from the aquarium biofilter, activated sludge (AS) and the rainwater tank (RT) samples affiliate with clade A Comammox, whereas sequences from soils (FI, CS) represent members of the clade B (Supplementary Fig. S2). A screening of lake water samples (n = 5) and samples of the sediment/water-interface (n = 9) from PIB with the equimolar primer mixture of Pjevac et al*.*^11^ (Table 1), originally designed for the specific detection of clade A Comammox, failed to produce PCR products. Additional investigations carried out with non-lake environmental samples produced specific amplification products of the expected length with DNA extracts obtained from RT and CS (Supplementary Fig. S3). Sequence analyses showed that sequences obtained from the RT and CS were affiliated with Comammox clade A *amoA* sequences (Supplementary Fig. S4). In order to enhance the sensitivity of the PCR assay, the number of PCR cycles was increased to 40 cycles and lake water samples (n = 49) were screened again. This enabled the generation of PCR products with DNA extracts from PIB, however the agarose gel revealed that several samples had off-target products in addition of amplicons of proper length (Supplementary Fig. S5). Sequence analysis of PCR products showed that eight out of 22 sequences were related to Comammox clade A *amoA* sequences (Supplementary Fig. S6), whereas all other off-target products had mostly a length of 768 bp and an unknown sequence identity. A thermal gradient experiment with DNA isolated from the RT analysed with droplet digital PCR also showed that it is not possible to separate unspecific PCR products based on the fluorescence amplitudes of the droplets (Supplementary Fig. S7). The primer mixture of Pjevac et al*.*^11^ (Table 1) targeting Comammox clade B sequences generated no PCR product, neither in lake water samples (n = 40) nor in other environmental samples. These findings did not correspond with the positive results obtained with the Ntsp-amoA 162F/359R^15^ primer pair (see above and Supplementary Fig. S2), suggesting that the clade B Comammox primer mixture has a limited coverage in these habitats. The primer sets of Beach and Noguera^16^, which were designed to specifically amplify the three Comammox Candidatus species *Nitrospira inopinata*, *Nitrospira nitrificans* and *Nitrospira nitrosa* (Table 1), did not produce amplification products in lake water samples (n = 37) and samples of the SWI (n = 9). Screening other environmental samples, a successful amplification was only achieved with the primer pair targeting *Ca. N. nitrificans* in the RT sample (Supplementary Fig. S8). Bartelme et al*.*^12^ designed a Comammox specific reverse primer, which is used in combination with a previous published forward primer targeting a gene encoding the alpha subunit of the particulate methane monooxygenase of methanotrophic prokaryotes^34^ (Table 1). This primer set produced only multiple off-target amplification products in one sample out of nine tested from SWI. Sequencing of PCR products with proper length showed that all sequences (n = 13) were not affiliated with Comammox. Taken together, all evaluated primer sets produced unspecific amplification products with exception of the species-specific primer sets^16^. However, the latter primers did not amplify *amoA* Comammox genes in any of the lake water samples. As the quantification of Comammox via qPCR is dependent on primers with a high specifity, non-specific amplification could result in an overestimation of *Nitrospira amoA* copy numbers. In particular the generation of off-target products with lengths almost identical to the targeted amplification products does not allow to separate these amplicons based on fluorescence amplitudes generated by ddPCR.

**Table 1:** Specification of primers used for Comammox *amoA* detection and quantification.

Target	Primer name	Sequence (5′–3′)	Product length (bp)	Ta^a^	References	Comments
Comammox *amoA* clade A	comaA-244f_a	ACA ACT GGG TGA ACT A	415	52	Pjevac et al*.*^11^	Equimolar mixture
	comaA-244f_b	TAT AAC TGG GTG AAC TA				
	comaA-244f_c	TAC AAT TGG GTG AAC TA				
	comaA-244f_d	TAC AAC TGG GTC AAC TA				
	comaA-244f_e	TAC AAC TGG GTC AAT TA				
	comaA-244f_f	TAT AAC TGG GTC AAT TA				
	comaA-659r_a	AGA TCA TGG TGC TAT G				
	comaA-659r_b	AAA TCA TGG TGC TAT G				
	comaA-659r_c	AGA TCA TGG TGC TGT G				
	comaA-659r_d	AAA TCA TGG TGC TGT G				
	comaA-659r_e	AGA TCA TCG TGC TGT G				
	comaA-659r_f	AAA TCA TCG TGC TGT G				
Comammox *amoA* clade B	comaB-244f_a	TAY TTC TGG ACG TTC TA	415	52	Pjevac et al*.*^11^	Equimolar mixture
	comaB-244f_b	TAY TTC TGG ACA TTC TA				
	comaB-244f_c	TAC TTC TGG ACT TTC TA				
	comaB-244f_d	TAY TTC TGG ACG TTT TA				
	comaB-244f_e	TAY TTC TGG ACA TTT TA				
	comaB-244f_f	TAC TTC TGG ACC TTC TA				
	comaB-659r_a	ARA TCC AGA CGG TGT G				
	comaB-659r_b	ARA TCC AAA CGG TGT G				
	comaB-659r_c	ARA TCC AGA CAG TGT G				
	comaB-659r_d	ARA TCC AAA CAG TGT G				
	comaB-659r_e	AGA TCC AGA CTG TGT G				
	comaB-659r_f	AGA TCC AAA CAG TGT G				
Comammox *amoA*	Ntsp-amoA 162F	GGA TTT CTG GNT SGA TTG GA	197	48	Fowler et al.^15^	
	Ntsp-amoA 359R	WAG TTN GAC CAC CAS TAC CA				
Comammox *amoA*	comaA1_336F	GTG GTG GTG GTC KAA CTA YCC	161	62	This study	
	comaA2_497R	GCC AGT TGC TCG GAT ARA AC				
Comammox *amoA*	pmoA-189b	GGN GAC TGG GAC TTY TGG	520	56	Luesken et al*.*^34^	
	Com_amoA_1_R	CGA GAT CAT GGT GCT GTG AC			Bartelme et al*.*^12^	
*Ca. Nitrospira inopinata amoA*	amoA-410F	TCA CCT TGT TGC TAA CTA GAA ACT GG	406	64	Beach and Noguera^16^	
	amoA-815R	TCC GCG TGA GCC AAT GT				
*Ca. Nitrospira nitrificans amoA*	amoA-463F	ATG TTC GCG GCA CTG TT	374			
	amoA-836R	CCA GAA AGT TTA GCT TTG TCG CCT				
*Ca. Nitrospira nitrosa amoA*	amoA-469F	GCG ATT CTG TTT TAT CCC AGC AAC	344			
	amoA-812R	CCG TGT GCT AAC GTG GCG				

^a^Ta: Annealing temperature (°C).

### Newly designed primer set for the quantification of Comammox in freshwater lakes

**Figure 1:**
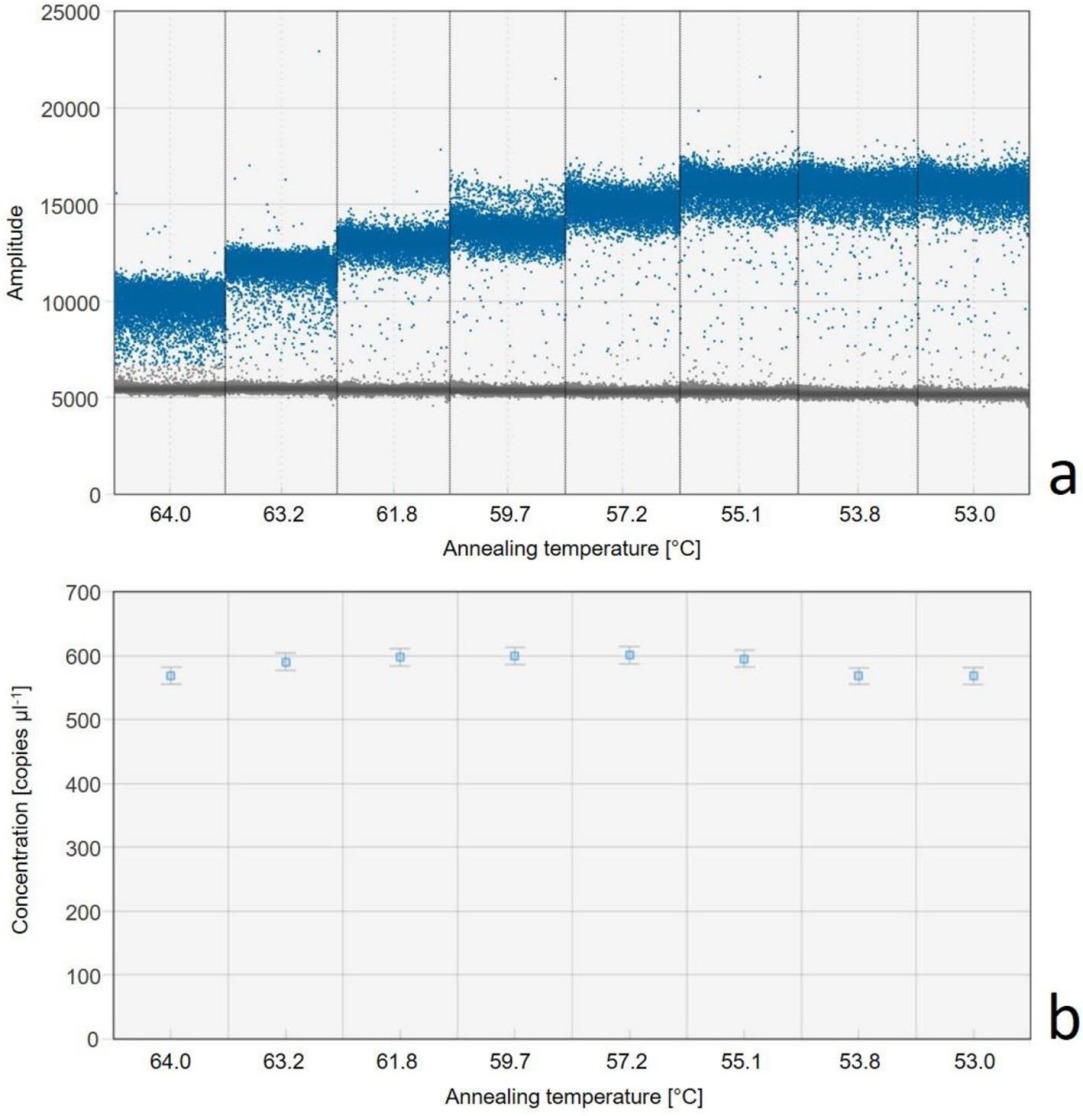
DdPCR thermal gradient experiment with a rainwater tank sample and the newly designed primer set ComaA1A2_336F/497R. (**a**) Shows the fluorescence amplitude plotted against the annealing temperature gradient. Positive droplets with PCR products are indicated in blue, negative droplets without any amplification are outlined grey. (**b**) Shows the corresponding concentrations along the temperature gradient, the error bars indicate 95% Poisson confidence interval.

**Figure 2:**
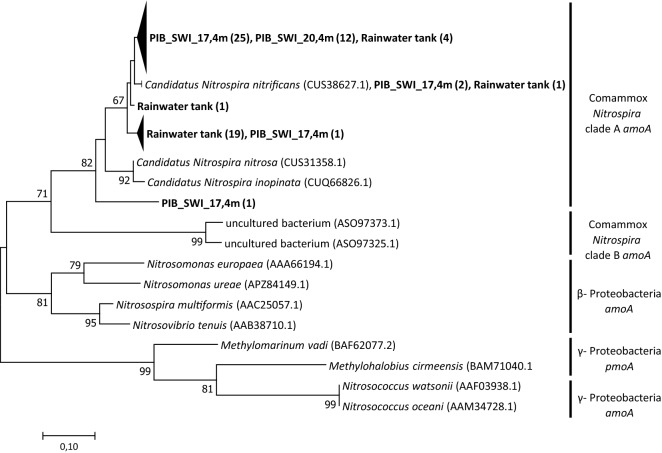
Neighbor-Joining tree of Comammox *amoA* sequences generated with the newly designed ComaA1A2_336F/497R primer set. The tree is based on deduced amino acid sequences of *amoA* genes obtained in this study (shown in bold) and representative reference sequences from NCBI database. Numbers in parentheses indicate the number of sequences. Bootstrap values are shown as percentages of 1000 replicates, values over 50% are indicated on nodes.

The primer pair ComaA1_336F (5′ GTG GTG GTG GTC KAA CTA YCC 3′) and ComaA2_497R (5′ GCC AGT TGC TCG GAT ARA AC 3′) was specifically designed to detect Comammox in lakes, targeting a cluster of sequences within the clade A of Comammox (Supplementary Fig. S9). The specificity of the newly designed primer pair based on potential mismatches with the targeted primer binding sites is shown in Supplementary Table S2. To test the suitability of this primer pair to produce a specific signal with the ddPCR, the optimal annealing temperature was determined by temperature gradient ddPCR experiments with a RT sample. Within an annealing temperature range from 53.0 to 64.0 °C, the primer pair revealed clearly distinguishable fluorescence signals up to an annealing temperature of 61.8 °C (Fig. 1). While at low annealing temperatures the chance of mispriming increases^35^, a consistent number of *amoA* copies over a wide range of annealing temperatures suggests a highly specific amplification of the targeted gene (Fig. 1). Therefore, for further investigations an annealing temperature of 62 °C was chosen. PCR analysis resulted in amplification products of proper length (161 bp) in samples from the SWI and RT. The taxonomic assignment of sequences (n = 66) obtained from the SWI and RT samples also confirmed that all sequences were affiliated with Comammox *amoA* sequences (Fig. 2). In some lake water samples droplets with a lower fluorescence intensity were generated by ddPCR, indicating the formation of primer dimers (Supplementary Fig. S10). However, a manual threshold setting enabled the exclusion of droplets with lower fluorescence intensity. Additional testing of aquatic samples (lake water, SWI and RT) based on the ddPCR resulted in a clearly distinguishable fluorescence signal with DNA extracts, also from samples characterised by low droplet counts (Supplementary Fig. S11). In contrast to the other primers tested, the newly designed primer pair enabled also the specific detection of Comammox in the samples of the SWI.

### Distribution of Comammox in lakes

**Figure 3:**
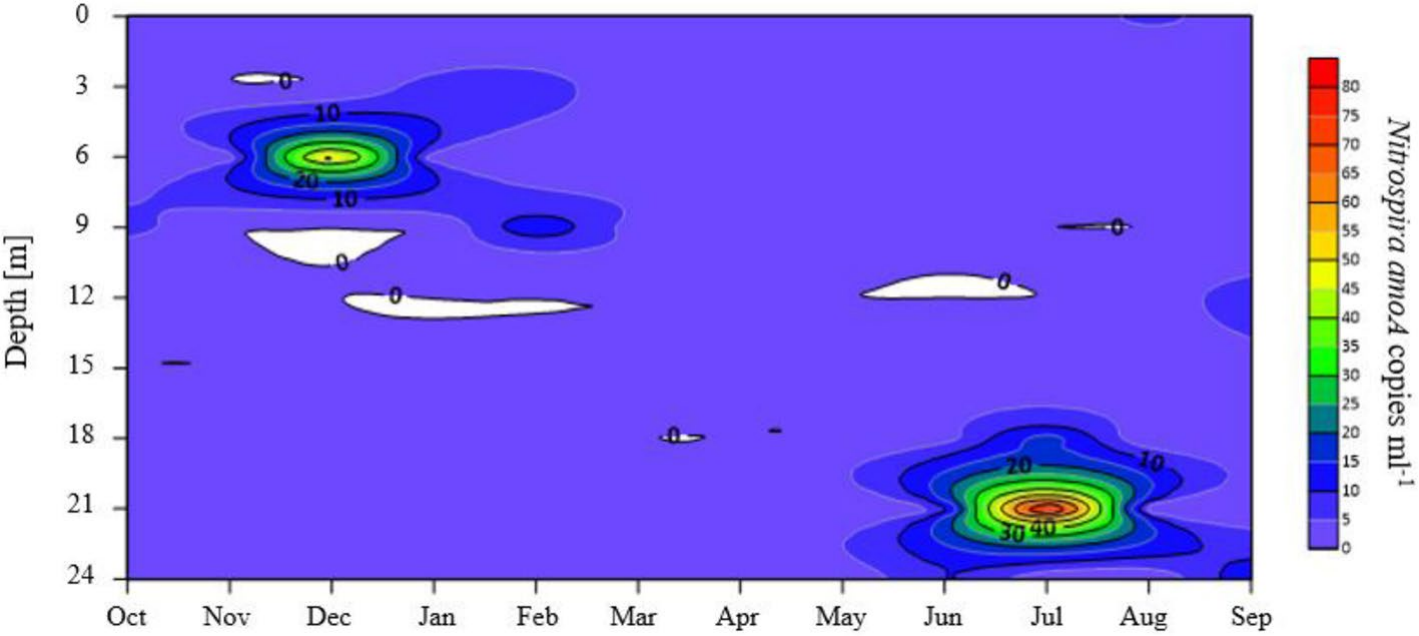
Spatiotemporal dynamics of Nitrospira *amoA* gene copy numbers in Piburger See. Samples were taken monthly with a vertical distance of 3 m.

Lakes sampled once during summer stagnation showed very low numbers of *Nitrospira amoA* close to the detection limit, not exceeding 17 *amoA* copies ml^−1^ (Supplementary Figs. S12 and S13). In lakes with a depth of less than 100 m the highest copy number was detected in EGE at 10 m depth. In deep lakes, highest *Nitrospira amoA* copy numbers were reached in ATT at 100 m. Data from the annual sampling campaign in PIB showed two maxima of *amoA* gene copies (Fig. 3). Samples taken in December at 6 m and July at 21 m reached the highest copy number, with 55.2 and 85.6 *Nitrospira amoA* copies ml^−1^, respectively. Taken all results together, the low abundances of Comammox *amoA* suggest a minor contribution of Comammox to the nitrification in the water column of the lakes investigated.

Based on data derived from substrate competition kinetics, Ca. *N. inopinata* showed low maximum oxidation rates, higher growth rates per mol ammonia und a higher ammonia affinity as all AOB and most AOA examined so far^36^. The high adaption of Comammox-*Nitrospira* to oligotrophic conditions is also reflected by the pathway Comammox is using to fulfil the carbon requirements. All *Nitrospira* use the reductive tricarboxylic acid cycle (rTCA) to assimilate inorganic carbon, a pathway that is considered to be far more energy efficient than other CO_2_ fixation cycles employing nonreducing carboxylases^37,38^. Considering these physiological characteristics, the central question raised by our results is why are Comammox rare in the pelagic water layers of the studied lakes? A wide range of different physicochemical conditions and generally low substrate concentrations would support the oligotrophic lifestyle of Comammox in lakes. Based on gene surveys in freshwater systems, Thaumarchaeota are the most important ammonia oxidizers in the water column of deep and oligotrophic lakes^25–27,39–43^. The dominance of AOA in the hypolimnion of deep lakes was already shown for lakes ACH, ATT, HAL, MIL, TRA, and ZUR, which were also investigated in the present study^25,26^. Due to the slow growth of Comammox^36^, they probably might be outcompeted by fast-growing canonical ammonia oxidizers including AOA. In contrast to deep lakes, nitrifiers in small lakes often show a high taxonomic heterogeneity among lakes and different seasons. AOA are often rare^25,26,33^. The absence of Comammox in the epilimnion of small lakes can be explained by high grazing pressure, competition for ammonia with phototrophs and light inhibition, factors that were already described as the best explanatory variables for the lack of AOP in the upper water layer in lakes^26,44,45^. This is also in agreement with the spatiotemporal distribution pattern of Comammox *amoA* in PIB, with highest numbers observed in December under ice and July in the hypolimnion of the lake.

**Figure 4:**
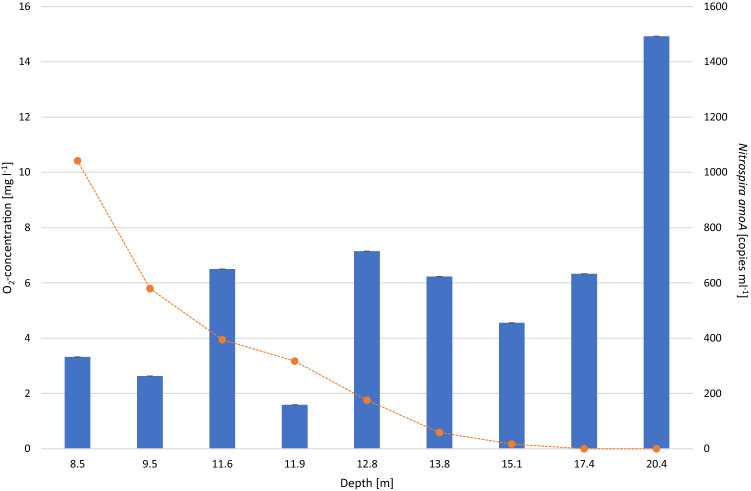
Concentration of dissolved oxygen (orange line) and *Nitrospira amoA* gene copy numbers (blue bars) in samples of the sediment/water-interface at different depths. Error bars indicate 95% Poisson confidence intervals.

Studies in engineered systems analysing biofilters and groundwater-fed rapid sand filters showed that Comammox-*Nitrospira* exceeded canonical ammonia oxidizers^12,15^. It was hypothesized that the higher number of Comammox compared to canonical ammonia oxidizers is mainly caused by one factor: these systems provide a suitable habitat for surface-attached microbial populations, in which the higher growth yield represents an advantage for Comammox^36,46,47^. This is also in agreement with studies that reported high numbers of *Nitrospira* in the interface of biofilms^48^, the tendency of aggregate formation in isolates^49,50^ and increasing abundances of Comammox-*Nitrospira* with an increasing retention time of aggregates in bioreactors^51^. In order to test the preferences of Comammox for a particle-attached lifestyle in lake water samples, the sediment/water-interface obtained along a transect characterized by different oxygen concentrations in PIB were investigated for the presence of Comammox. All nine samples of this sediment-overlying water layer were rich in particles, providing a suitable habitat for microbial surface colonization. *Nitrospira amoA* copy numbers in the SWI were at least three orders of magnitude higher than in all pelagic water samples, with a maximum of 0.149 × 10^6^ copies ml^−1^ at 20.4 m depth (Fig. 4). Interestingly, *amoA* copy number did not correlate with the oxygen concentrations and the highest copy numbers were detected in 20.4 m depth, where DO was not detectable (Fig. 4). In this oxygen-free environment, Comammox would have to use alternative metabolisms for energy conservation, which has already been demonstrated for nitrite-oxidizing bacteria from the genus *Nitrospira*^52^. For example, it was shown that *Nitrospira moscoviensis* oxidize formiate with nitrate as terminal electron acceptor in the absence of oxygen^52^. Another aspect that has to be considered is that determining anoxia based exclusively on standard oxygen measurements could be biased by technical limitation. All oxygen measurements in the SWI were done with a multi-parameter probe equipped with an optical DO sensor, which is limited to detect oxygen concentrations < 1 µM. However, it was already shown for oxygen minimum zone waters in marine systems that the two key aerobic nitrifications processes, ammonia oxidation and nitrite oxidation, persist even at very low oxygen levels of 5–30 nM^53,54^.

## Conclusion

Primer coverage and specificity poses a permanent problem for the accurate quantification of most functional genes obtained from environmental samples, especially when targeting genes coding for key enzymes in different nitrifying organism. Often, a degenerated primer set or multiple primers are necessary to cover highly divergent gene diversity within the same functional groups. In this study, a major effort was undertaken to evaluate already published PCR-based marker systems. These primers generated non-specific PCR products or did not amplify *amoA* Comammox genes in any of the lake water samples tested. Therefore, we designed a novel primer set that enabled quantification of Comammox *Nitrospira* by droplet digital PCR with high coverage and specificity in lake water, but also in samples from other habitats. Using this primer set for quantification of Comammox *amoA* genes in the water column of 13 lakes revealed very low abundances, which suggest that Comammox is of minor importance for the nitrification process in the pelagic environment. However, samples taken from the benthic boundary layer of one of the lakes investigated were highly populated by Comammox. We hypothesize that this particle-rich environment provides a potential habitat for microbial surface colonization, suggesting the preference of Comammox for particle-attachment. Additional research is certainly needed to determine niche preferences of Comammox-*Nitrospira* in lakes, focusing on habitats characterized by different trophic states and oxygen gradients, but also to test in detail if Comammox favors a surface-attached lifestyle.

## Methods

### Study sites, sampling and DNA-extraction

For this study, pelagic water samples from different depths of 13 lakes (Achensee—ACH, Attersee—ATT, Egelsee—EGE, Faakersee—FAA, Hallstättersee—HAL, Hechtsee—HEC, Irrsee—IRR, Millstätter See—MIL, Mondsee—MON, Piburger See—PIB, Traunsee—TRA, Weißensee—WEI, Zürichsee—ZUR) with a wide range of physicochemical properties were analysed. The geographical location, sampling regimes, key limnological and chemical parameters of the lakes are described in detail elsewhere^25,26,33^. Samples of the annual cycle in Piburger See were collected on a monthly basis in a former study^33^, along a vertical profile of 3 m intervals at the deepest part of the lake (24.6 m). All lake water samples were taken with a modified Schindler-Patalas sampler (UWITEC GmbH, Mondsee, Austria). Prior to sampling, measurements of dissolved oxygen were performed with a multi-parameter probe (YSI model 6600, Yellow Springs Instruments, Ohio, USA). To evaluate primer pairs that failed to produce an amplification product with lake water samples, non-lake habitat samples were collected and analysed: Biofilter (BF) samples were obtained by squeezing out filter sponges of an external freshwater fish tank biofilter which operates at a temperature of ~ 26 °C. For the rainwater tank (RT) sample, the biofilm developed on the surface of a rain barrel was sampled. Activated sludge (AS) samples were obtained from a wastewater treatment plant. A mud sample of a pond (PO) with a high fish density was collected. The terrestrial samples encompass the topsoil layer of a maize field (FI) and compost soil (CS). The sediment/water-interface (SWI) of Piburger See was sampled at the northwestern lake basin along a depth gradient from 8.5 to 20.4 m and processed as follows: samples were vortexed vigorously for 10 s and an aliquot of 45 ml volume was transferred into 50 ml tubes and stored at − 20 °C until further use. For DNA-extraction the supernatant was removed after a sedimentation time of 30 min, the remaining aggregates were vortexed vigorously for 10 s. 250 µl of the sample were transferred for further DNA-extraction. DNA-extraction of SWI samples and other non-lake environmental samples was performed with a DNeasy PowerMax Soil Kit (QIAGEN Inc., Hilden, Germany) following the manufacturer’s instructions. Sample processing and DNA extraction of pelagic water samples are described elsewhere^25,26,33^. DNA-concentrations were measured fluorometrically (Quantus Fluorometer and QuantiFluor dsDNA chemistry, Promega Corporation, Fitchburg, USA).

### Primer evaluation and design, PCR and sequencing analysis

To evaluate the specifity and coverage of previously published primers (Table 1) targeting Comammox *amoA*, PCR analysis were applied using DNA-extracts of samples obtained from different lakes. If primers failed to produce an amplicon of proper length, additional PCRs were carried out with DNA-extracts from other environmental samples. PCRs were performed with a DreamTaq PCR Master Mix (Thermo Scientific Inc., Waltham, USA) in a total volume of 15 µl (7.5 µl Mastermix (2×), 1 µl DNA-template, 1 µl of each Reverse/Forward Primer (10 µM), 4.5 µl nuclease-free water) following the manufacturer’s instructions. DNA-extracts of pelagic water samples were used in 1:10 dilutions due to the presence of potential PCR inhibitory compounds. Annealing temperatures used for individual primer sets are listed in Table 1. For sequence generation, 1 µl of the PCR-reactions was separated on 1.5% agarose gels. Amplicons of expected length were cut out and purified using MinElute Gel Extraction Kit (QIAGEN Inc.). Purified PCR-products were ligated into pGEM-T-Easy Vector plasmids (Promega Corporation) and heat-shock transformed into JM109 competent cells. Colony PCRs were performed with randomly picked white colonies using vector-specific M13-F/R Primers and DreamTaq PCR Master Mix (Thermo Scientific Inc.) according to the protocol of the manufacturer followed by visual inspection of DNA bands on an agarose gel. Selected insert containing PCR-products were Sanger sequenced by a sequencing service enterprise (Eurofins MWG Operon, Ebersberg, Germany).

### Phylogenetic analysis and design of Comammox specific primers

The EMBOSS Transeq tool^55^ was used to translate nucleic acid sequences to their corresponding amino acid sequences. Closest relatives to nucleotide sequences and deduced amino acid sequences were obtained using NCBI BLASTN and BLASTP search tool^56^. Sequences were aligned using MUSCLE algorithm integrated in MEGA 6.0 software^57^ and the alignment of the deduced amino acid sequences was checked visually. For phylogenetic reconstruction Neighbor-Joining algorithm (MEGA 6.0 software) was applied, the trees were calculated with gamma distribution as distance method followed by a bootstrapping analysis with 1000 replicates to estimate the robustness of tree topology. For the design of the new primer pair targeting the Comammox clade A cluster, sequences obtained from Piburger See, the rainwater tank and selected sequences retrieved from the NCBI Genbank database, were analysed (see Supplementary Information Fig. S9). Sequences were screened for conserved regions as potential primer binding sites using Primer-BLAST^58^ and Jalview 2 software^59^. To avoid the formation of primer dimers and to check the compatibility of possible primer candidates, NetPrimer analysis software (http://www.premierbiosoft.com/netprimer/) was used. The final in silico analysis of primer coverage was performed using Primer BLAST^58^.

### Droplet digital PCR

For quantification of gene copy numbers, a ddPCR system was used (QX200, Bio-Rad Laboratories, Hercules, USA). DdPCR-reactions were prepared in 96-well plates with QX200 ddPCR EvaGreen Supermix (Bio-Rad) with a final volume of 22 µl and a primer concentration of 100 nm according to the manufacturer’s protocol. Plates were sealed by heat and for droplet generation an automated droplet generator (AutoDG Instrument, Bio-Rad) was used. A three-step PCR amplification was performed with a ramp rate of 2 °C/s under the following cycling conditions: 3 min at 95 °C, 40 cycles including 30 s at 95 °C, 1 min at primer specific annealing temperature (see Table 1) and 2 min at 72 °C, followed by 5 min at 4 °C and 5 min at 90 °C for signal stabilization of the PCR. The optimal annealing temperature of the newly designed primer pair was determined based on temperature gradient experiments (Fig. 1). Final quantification of Comammox *amoA* gene copy numbers in lake water samples was performed using ComaA1A2_336F/497R primer set (Table 1). Fluorescence signals were measured using a droplet reader (QX200, Bio-Rad) according to the protocol of the manufacturer, subsequent analyses of data were performed using QuantaSoft Analysis Pro Software (Bio-Rad) with a final visual check to evaluate the reliability of the automated threshold settings.

### Sequence data deposition

All obtained sequences were deposited at NCBI Genbank and are available under accession numbers

MN684849-MN684894 and MN684984-MN685049.

## Supplementary Information

Supplementary Information.
