# Supplementary material for: Primer evaluation and development of a droplet digital PCR protocol targeting amoA genes for the quantification of Comammox in lakes
Source: Sci Rep. 2021 Feb 3;11:2982. doi: 10.1038/s41598-021-82613-6 (PMC7858572; doi:10.1038/s41598-021-82613-6)
Supplement: Supplementary file 1 — Supplementary Information. [file 41598_2021_82613_MOESM1_ESM.pdf]

Supplementary Informartion

Primer evaluation and development of a droplet digital PCR protocol targeting *amoA* genes for the quantification of Comammox in lakes

Manuel Harringer[1], Albin Alfreider[1]*

[1]Department of Ecology, University of Innsbruck, Innsbruck, Austria;

*Corresponding author Albin Alfreider: Tel: +43 512 507-51732

E-mail: albin.alfreider@uibk.ac.at

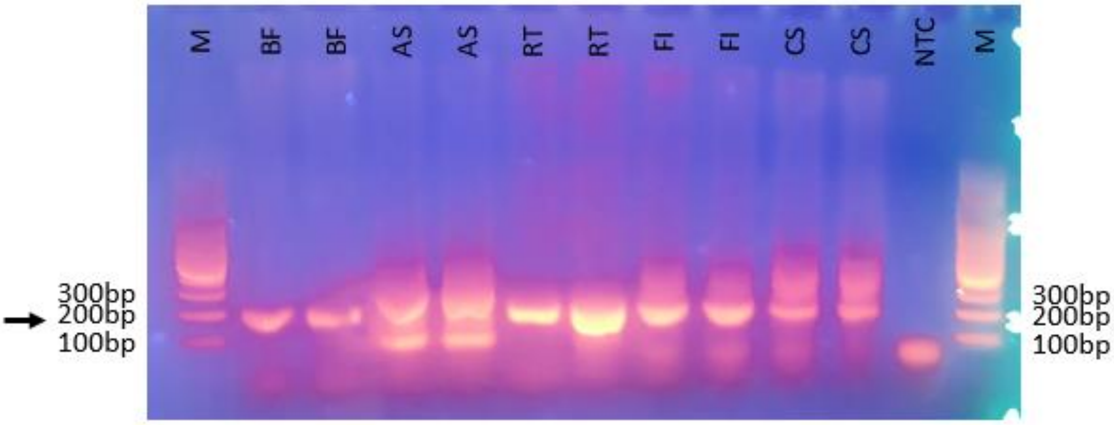

Figure S1: Display of full-length images of agarose gel electrophoresis with PCR products (includes one replicate) using Ntsp-amoA 162F/359R primer set (primer details are given in Table 1). The arrow on the left side shows the expected amplicon size. M= 100 bp DNA size marker, BF= biofilter, AS= activated sludge, RT= rainwater tank, FI= field, CS= compost soil, NTC= non template control;

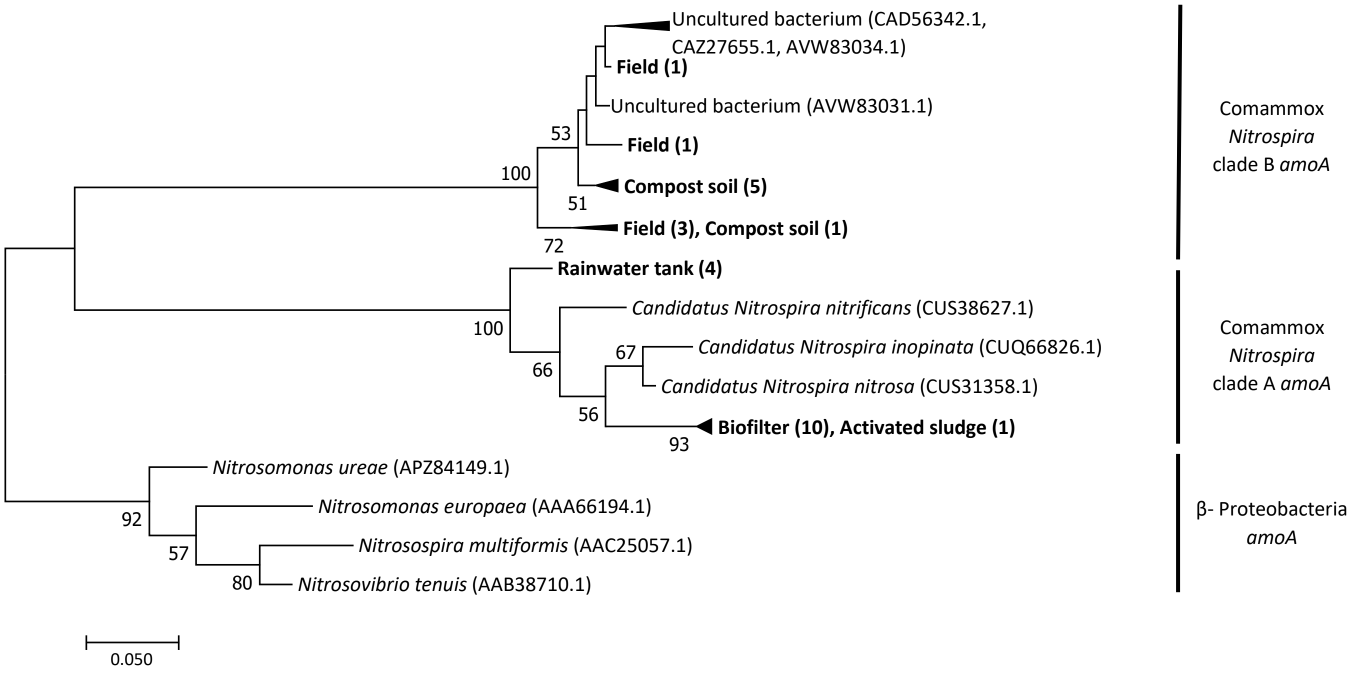

Figure S2: Neighbor-Joining tree of Comammox *amoA* sequences generated with the Ntsp-amoA 162F/359R primer set (primer details are given in Table 1). The tree is based on deduced amino acid sequences of *amoA* genes from this study (shown in bold) and representative reference sequences from the NCBI database. Numbers in parentheses indicate the number of sequences. Bootstrap values are shown as percentages of 1000 replicates, values over 50% are indicated on nodes.

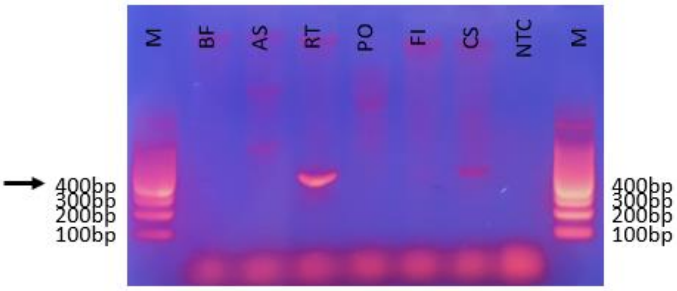

Figure S3: Display of full-length images of agarose gel electrophoresis with PCR products using the equimolar clade A primer mixture (primer details are given in Table 1). The arrow on the left side shows the expected amplicon size. M= 100 bp DNA size marker, BF= biofilter, AS= activated sludge, RT= rainwater tank, PO= pond, FI= field, CS= compost soil, NTC= non template control;

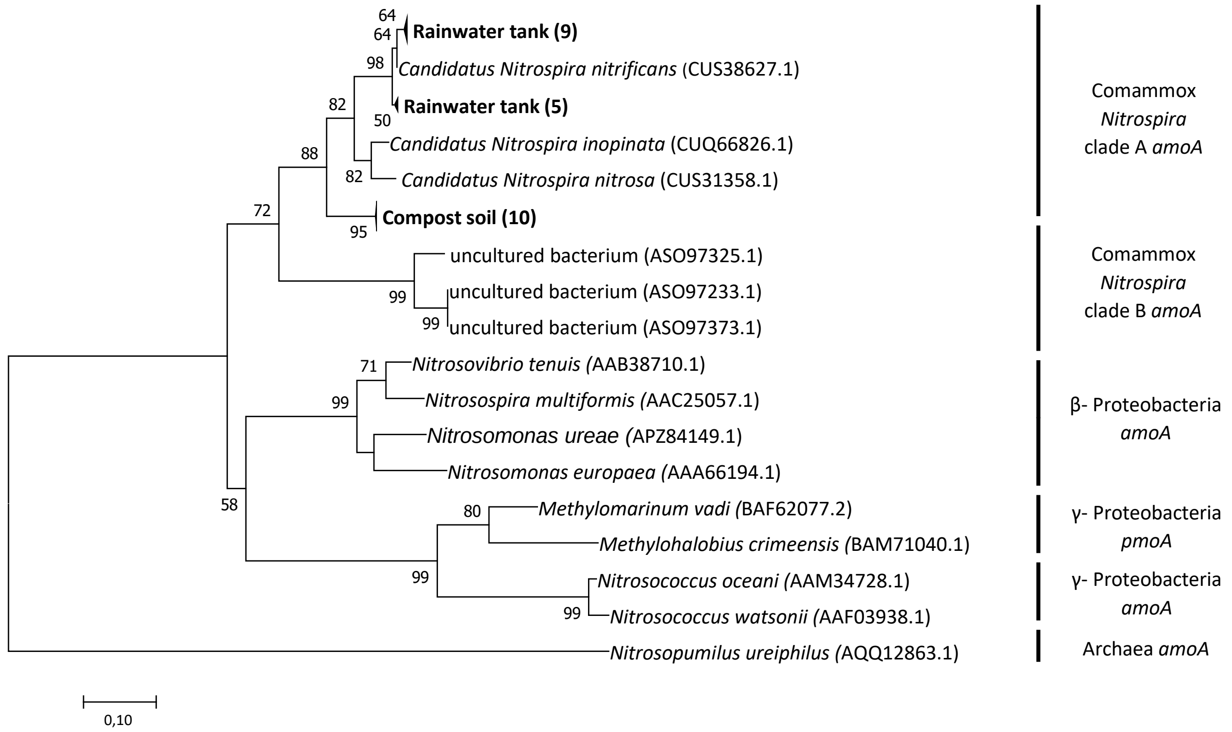

Figure S4: Neighbor-Joining tree of Comammox *amoA* sequences generated with the equimolar clade A primer mixture (primer details are given in Table 1). The tree is based on deduced amino acid sequences of *amoA* genes from this study (shown in bold) and representative reference sequences from the NCBI database. Numbers in parentheses indicate the number of sequences. Bootstrap values are shown as percentages of 1000 replicates, values over 50% are indicated on nodes.

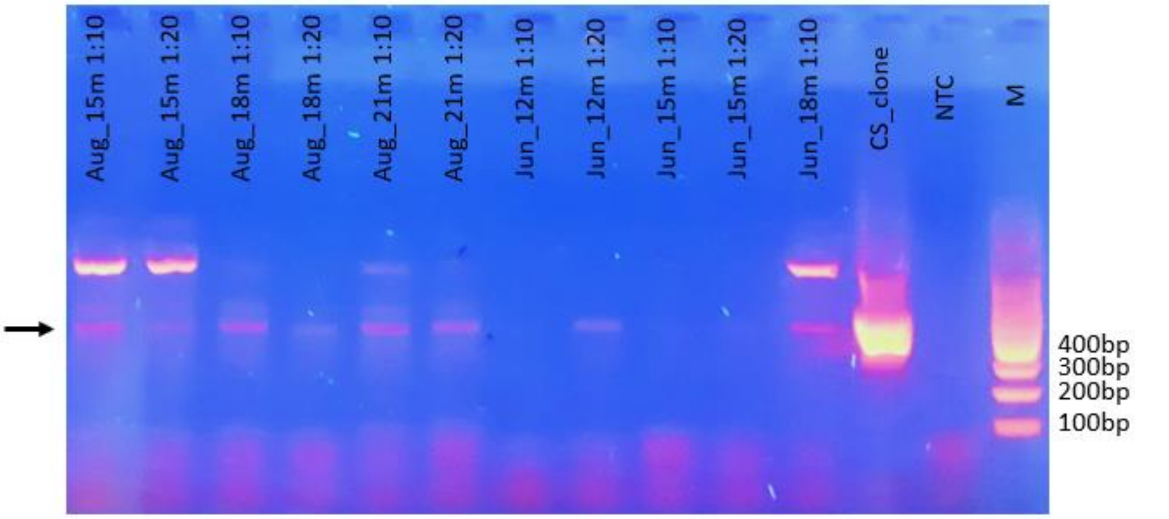

Figure S5: Display of full-length images of agarose gel electrophoresis with PCR products using the equimolar clade A primer mixture (primer details are given in Table 1) with diluted lake water samples of Piburger See. The arrow on the left side shows the expected amplicon size. CS_clone= compost soil clone, NTC= non template control, M= 100 bp DNA size marker;

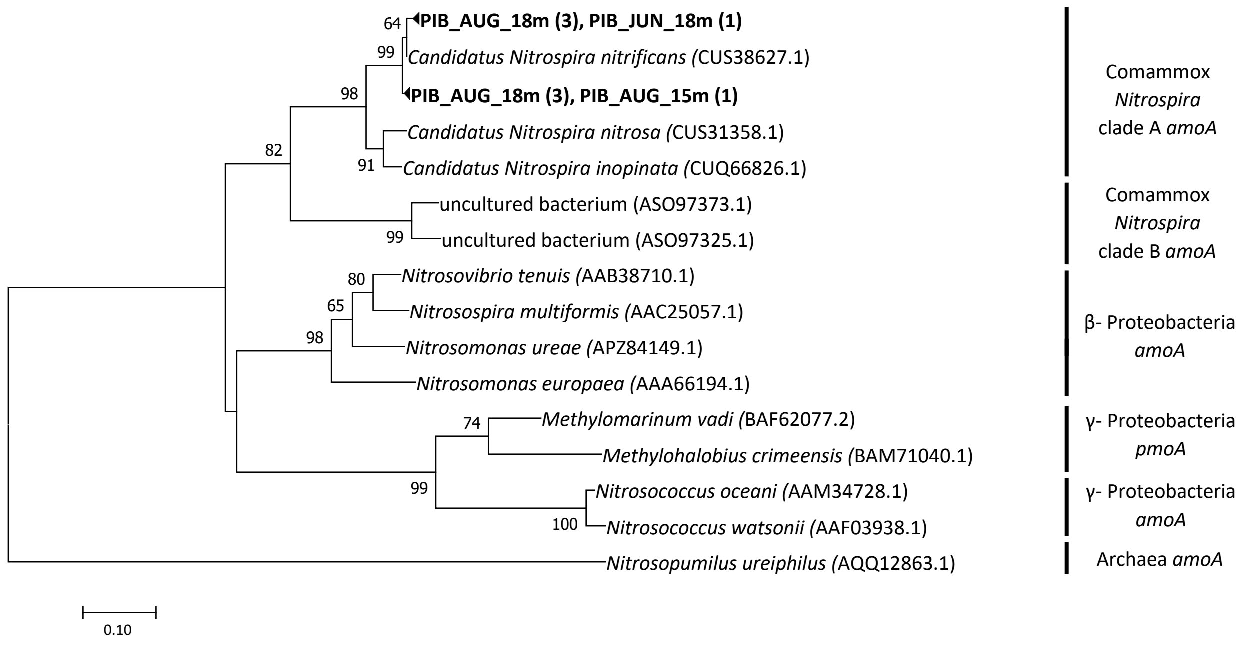

Figure S6: Neighbor-Joining tree of Comammox *amoA* sequences generated with the equimolar clade A primer mixture (primer details are given in Table 1). The tree is based on deduced amino acid sequences of *amoA* genes from Piburger See (shown in bold) and representative reference sequences from NCBI database. Numbers in parentheses indicate the number of sequences. Bootstrap values are shown as percentages of 1000 replicates, values over 50% are indicated on nodes.

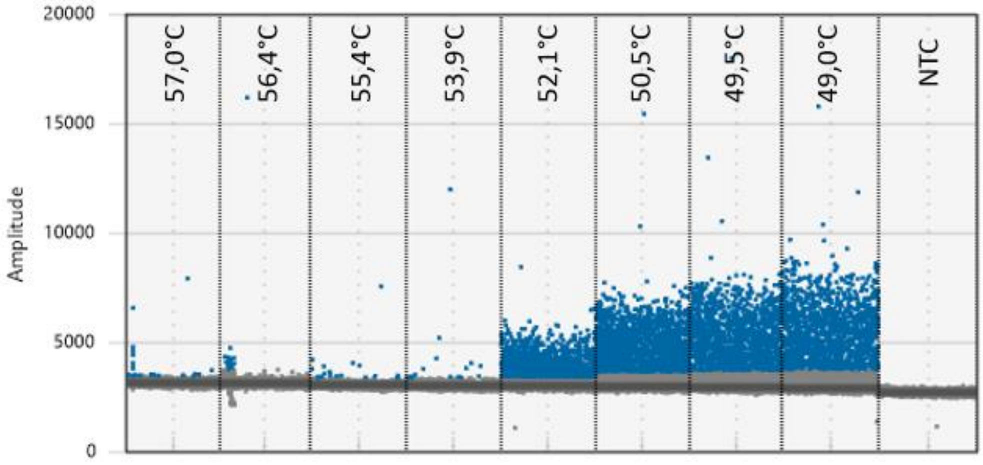

Figure S7: Thermal gradient ddPCR experiment with the equimolar clade A primer mixture (primer details are given in Table 1) and DNA extracts of the rainwater tank as template. The figure shows positive (blue) and negative (grey) droplets based on their fluorescence amplitude (y-axis). NTC= non template control.

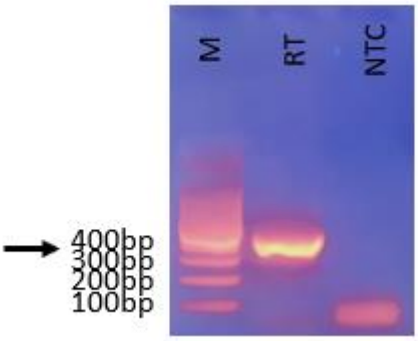

Figure S8: Display of full-length images of agarose gel electrophoresis with PCR products using the *Candidatus Nitrospira nitrificans* specific primer set (primer details are given in Table 1). The arrow on the left side shows the expected amplicon size. M= 100 bp DNA size marker, RT= rainwater tank, NTC= non template control;

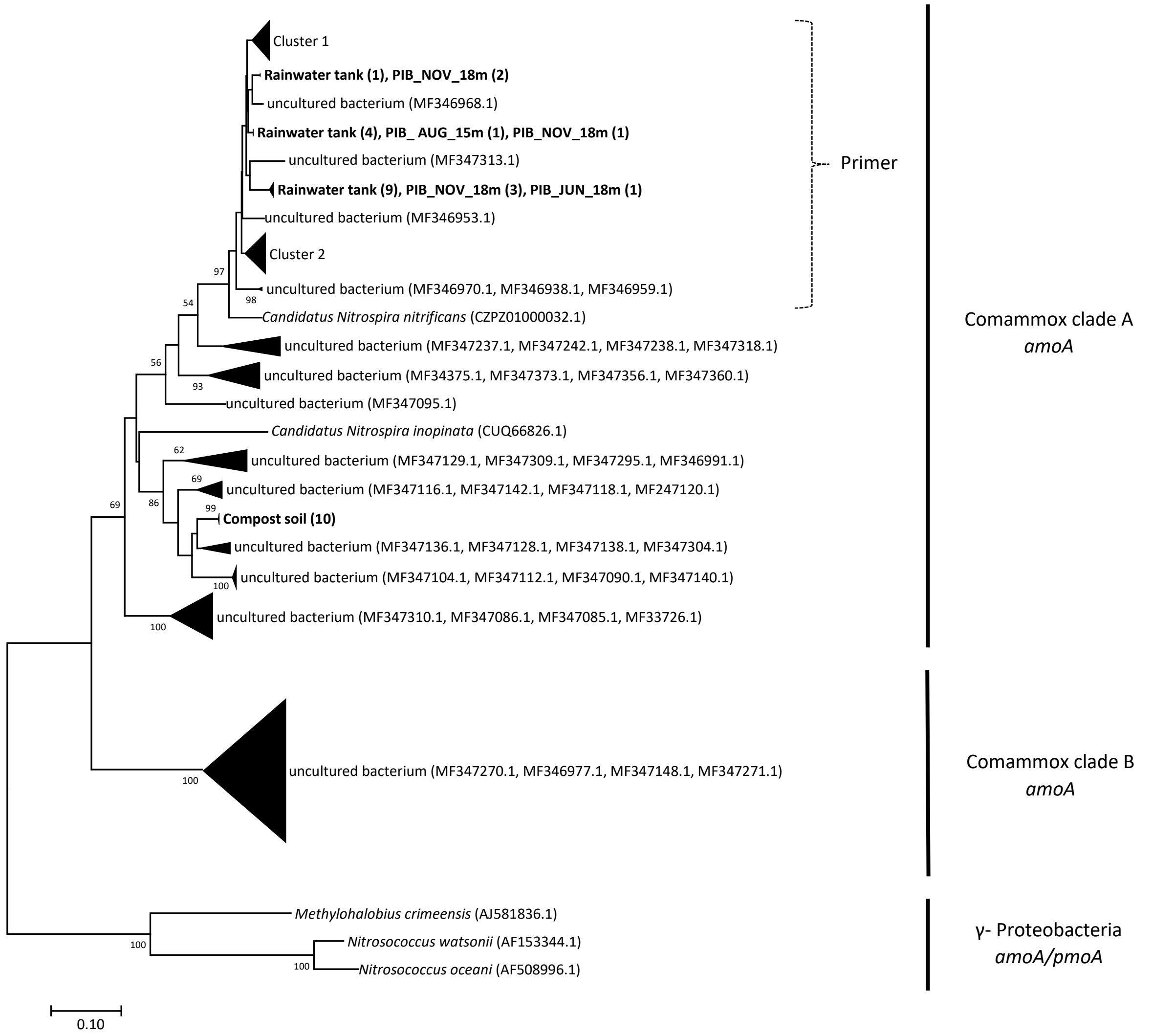

Figure S9: Coverage of the new primer pair including sequences used for the design of the new primer set. The Neighbor Joining tree is based on nucleic acid sequences of *amoA* genes from this study derived with Comammox clade A primer mixture (shown in bold, primer details are given in Table 1) and representative reference sequences from the NCBI database. Numbers in parentheses indicate the number of sequences. For each condensed cluster outside of the primer cluster (indicated by the dashed bracket) the accession numbers of four randomly chosen representative sequences are given. The accession numbers of "Cluster 1" and "Cluster 2" within the primer cluster are given in Table S1 below. Bootstrap values are shown as percentages of 1000 replicates, values over 50% are indicated on nodes. Sequences of primer binding sites within the primer cluster are shown in Table S2 below.

Table S1: Accession numbers of sequences in "Cluster 1" and "Cluster 2" of figure S9.

Cluster 1		Cluster 2	
MF347001.1	MF347025.1	MF347000.1	MF346962.1
MF347006.1	MF347026.1	MF347002.1	MF346963.1
MF347009.1	MF347027.1	MF347003.1	MF346964.1
MF347010.1	MF347028.1	MF347004.1	MF346966.1
MF347011.1	MF347029.1	MF347005.1	MF346967.1
MF347012.1	MF347030.1	MF347007.1	MF346969.1
MF347013.1	MF347300.1	MF347008.1	MF346989.1
MF347014.1	MF347303.1	MF346936.1	MF346992.1
MF347015.1	MF347306.1	MF346937.1	MF346993.1
MF347016.1	MF347307.1	MF346939.1	MF346994.1
MF347017.1	MF347312.1	MF346940.1	MF346995.1
MF347018.1	MF347314.1	MF346954.1	MF346996.1
MF347019.1	MF347362.1	MF346955.1	MF346997.1
MF347020.1	MF346988.1	MF346956.1	MF346999.1
MF347021.1	MF346965.1	MF346957.1	MF347301.1
MF347022.1	MF347323.1	MF346958.1	MF347302.1
MF347023.1		MF346960.1	MF346961.1
MF347024.1		MF346971.1	

Table S2. Primer binding sites based on sequences of *amoA* genes of the primer cluster shown in Figure S9. Red letters indicate mismatches to newly designed primer pair ComaA1_336F/ ComaA2_497R.

Genbank Accession Number	Forward Primer Binding Sites (5'-3')	Reverse Primer Binding Sites (5'-3')
MN684858.1	GTGGTGGTGGTCTAACTATCC	GTTCTATCCGAGCAACTGGC
MN684859.1	GTGGTGGTGGTCTAACTATCC	GTTCTATCCGAGCAACTGGC
MN684860.1	GTGGTGGTGGTCTAACTATCC	GTTCTATCCGAGCAACTGGC
MN684861.1	GTGGTGGTGGTCTAACTATCC	GTTCTATCCGAGCAACTGGC
MN684862.1	GTGGTGGTGGTCTAACTATCC	GTTCTATCCGAGCAACTGGC
MN684850.1	GTGGTGGTGGTCTAACTATCC	GTTCTATCCGAGCAACTGGC
MN684851.1	GTGGTGGTGGTCTAACTATCC	GTTCTATCCGAGCAACTGGC
MN684852.1	GTGGTGGTGGTCTAACTATCC	GTTCTATCCGAGCAACTGGC
MN684853.1	GTGGTGGTGGTCTAACTATCC	GTTCTATCCGAGCAACTGGC
MN684854.1	GTGGTGGTGGTCTAACTATCC	GTTCTATCCGAGCAACTGGC
MN684855.1	GTGGTGGTGGTCTAACTATCC	GTTCTATCCGAGCAACTGGC
MN684856.1	GTGGTGGTGGTCTAACTATCC	GTTCTATCCGAGCAACTGGC
MN684857.1	GTGGTGGTGGTCTAACTATCC	GTTTTATCCGAGCAACTGGC
MN684894.1	GTGGTGGTGGTCTAACTATCC	GTTTTATCCGAGCAACTGGC
MN684890.1	GTGGTGGTGGTCTAACTATCC	GTTCTATCCGAGCAACTGGC
MN684887.1	GTGGTGGTGGTCTAACTATCC	GTTCTATCCGAGCAACTGGC
MN684892.1	GTGGTGGTGGTCTAACTATCC	GTTCTATCCGAGCAACTGGC
MN684889.1	GTGGTGGTGGTCTAACTATCC	GTTTTATCCGAGCAACTGGC
MN684888.1	GTGGTGGTGGTCTAACTATCC	GTTCTATCCGAGCAACTGGC
MN684891.1	GTGGTGGTGGTCTAACTATCC	GTTCTATCCGAGCAACTGGC
MN684893.1	GTGGTGGTGGTCTAACTATCC	GTTCTATCCGAGCAACTGGC
CZPZ01000032.1	GTGGTGGTGGTCCAATTATCC	GTTCTATCCGAGCAACTGGC
MF346936.1	GTGGTGGTGGTCGAACTACCC	GTTCTATCCGAGCAACTGGC
MF346937.1	GTGGTGGTGGTCGAACTACCC	GTTCTATCCGAGCAACTGGC
MF346938.1	GTACTGGTGGTCAAATTATCC	GTTCTATCCGAGCAACTGGC
MF346939.1	GTGGTGGTGGTCTAACTATCC	GTTCTATCCGAGCAACTGGC
MF346940.1	GTACTGGTGGTCAAATTATCC	GTTCTATCCGAGCAACTGGC
MF346953.1	GTGGTGGTGGTCTAACTATCC	GTTCTATCCGAGCAACTGGC
MF346954.1	GTGGAGGTGGTCGAACTACCC	GTTCTATCCGAGCAACTGGC
MF346955.1	GTGGTGGTGGTCGAACTACCC	ATTCTATCCGAGCAACTGGC
MF346956.1	GTGGTGGTGGTCGAACTACCC	ATTCTATCCGAGCAACTGGC
MF346957.1	GTGGTGGTGGTCGAACTACCC	GTTCTATCCGAGCAACTGGC
MF346958.1	GTGGTGGTGGTCGAACTACCC	ATTCTATCCGAGCAACTGGC
MF346959.1	GTACTGGTGGTCAAATTATCC	GTTCTATCCGAGCAACTGGC
MF346960.1	GTGGTGGTGGTCGAACTACCC	ATTCTATCCGAGCAACTGGC
MF346961.1	GTGGTGGTGGTCGAACTACCC	GTTCTATCCGAGCAACTGGC
MF346962.1	GTGGTGGTGGTCGAACTACCC	ATTCTATCCGAGCAACTGGC
MF346963.1	GTGGTGGTGGTCTAACTATCC	GTTTTATCCGAGCAACTGGC
MF346964.1	GTGGTGGTGGTCGAACTACCC	ATTCTATCCGAGCAACTGGC
MF346965.1	GTGGTGGTGGTCCAACTATCC	GTTCTATCCGAGCAACTGGC
MF346966.1	GTGGTGGTGGTCGAACTACCC	ATTCTATCCGAGCAACTGGC
MF346967.1	GTGGTGGTGGTCGAACTACCC	GTTCTATCCGAGCAACTGGC
MF346968.1	GTGGTGGTGGTCTAACTATCC	GTTCTATCCGAGCAACTGGC
MF346969.1	GTGGTGGTGGTCGAACTACCC	ATTCTATCCGAGCAACTGGC
MF346970.1	GTACTGGTGGTCAAATTATCC	GTTCTATCCGAGCAACTGGC
MF346971.1	GTGGTGGTGGTCGAACTACCC	ATTCTATCCGAGCAACTGGC
MF346988.1	GTGGTGGTGGTCTAACTATCC	GTTCTATCCGAGCAACTGGC
MF346989.1	GTGGTGGTGGTCTAACTATCC	GTTCTATCCGAGCAACTGGC
MF346992.1	GTGGTGGTGGTCTAACTATCC	GTTCTATCCGAGCAACTGGC
MF346993.1	GTGGTGGTGGTCTAACTATCC	GTTCTATCCGAGCAACTGGC
MF346994.1	GTGGTGGTGGTCTAACTATCC	GTTCTATCCGAGCAACTGGC
MF346995.1	GTGGTGGTGGTCTAACTATCC	GTTCTATCCGAGCAACTGGC
MF346996.1	GTGGTGGTGGTCTAACTATCC	GTTCTATCCGGGCAACTGGC
MF346997.1	GTGGTGGTGGTCTAACTATCC	GTTCTATCCGAGCAACTGGC
MF346999.1	GTGGTGGTGGTCTAACTATCC	GTTCTATCCGAGCAACTGGC
MF347000.1	GTGGTGGTGGTCTAACTATCC	GTTCTATCCGAGCAACTGGC
MF347001.1	GTGGTGGTGGTCTAACTATCC	GTTCTATCCGAGCAACTGGC
MF347002.1	GTGGTGGTGGTCTAACTATCC	GTTCTATCCGAGCAACTGGC
MF347003.1	GTGGTGGTGGTCTAACTATCC	GTTCTATCCGAGCAACTGGC
MF347004.1	GTGGTGGTGGTCTAACTATCC	GTTCTATCCGAGCAACTGGC
MF347005.1	GTGGTGGTGGTCTAACTATCC	GTTCTATCCGAGCAACTGGC
MF347006.1	GTGGTGGTGGTCTAACTATCC	GTTCTATCCGAGCAACTGGC
MF347007.1	GTGGTGGTGGTCTAACTATCC	GTTCTATCCGAGCAACTGGC
MF347008.1	GTGGTGGTGGCCTAACTATCC	GTTCTATCCGAGCAACTGGC
MF347009.1	GTGGTGGTGGTCTAACTATCC	GTTCTATCCGAGCAACTGGC
MF347010.1	GTGGTGGTGGTCTAACTATCC	GTTCTATCCGAGCAACTGGC
MF347011.1	GTGGTGGTGGTCTAACTATCC	GTTCTATCCGAGCAACTGGC
MF347012.1	GTGGTGGTGGTCTAACTATCC	GTTCTATCCGAGCAGCTGGC
MF347013.1	GTGGTGGTGGTCTAACTATCC	GTTCTATCCGAGCAACTGGC
MF347014.1	GTGGTGGTGGTCTAACTATCC	GTTCTATCCGAGCAACTGGC
MF347015.1	GTGGTGGTGGTCTAACTATCC	GTTCTATCCGAGCAACTGGC
MF347016.1	GTGGTGGTGGTCTAACTATCC	GTTCTATCCGAGCAACTGGC
MF347017.1	GTGGTGGTGGTCTAACTATCC	GTTCTATCCGAGCAACTGGC
MF347018.1	GTGGTGGTGGTCTAACTATCC	GTTCTATCCGAGCAACTGGC
MF347019.1	GTGGTGGTGGTCTAACTATCC	GTTCTACCCGAGCAACTGGC
MF347020.1	GTGGTGGTGGTCTAACTATCC	GTTCTATCCGAGCAACTGGC
MF347021.1	GTGGTGGTGGTCTAACTATCC	GTTCTATCCGAGCAACTGGC
MF347022.1	GTGGTGGTGGTCTAACTATCC	GTTCTATCCGAGCAACTGGC
MF347023.1	GTGGTGGTGGTCTAACTATCC	GTTCTATCCGAGCAACTGGC
MF347024.1	GTGGTGGTGGTCTAACTATCC	GTTCTATCCGAGCAACTGGC
MF347025.1	GTGGTGGTGGTCTAACTATCC	GTTCTATCCGAGCAACTGGC
MF347026.1	GTGGTGGTGGTCTAACTATCC	GTTCTATCCGAGCAACTGGC
MF347027.1	GTGGTGGTGGTCTAACTATCC	GTTCTATCCGAGCAACTGGC
MF347028.1	GTGGTGGTGGTCTAACTATCC	GTTCTATCCGAGCAACTGGC
MF347029.1	GTGGTGGTGGTCTAACTATCC	GTTCTATCCGAGCAACTGGC
MF347030.1	GTGGTGGTGGTCTAACTATCC	GTTCTATCCGAGCAACTGGC
MF347300.1	GTGGTGGTGGTCTAACTATCC	GTTCTATCCGAGCAACTGGC
MF347301.1	GTGGTGGTGGTCTAATTATCC	GTTCTATCCAAGCAACTGGC
MF347302.1	GTGGTGGTGGTCTAACTATCC	GTTCTATCCGAGCAACTGGC
MF347303.1	GTGGTGGTGGTCTAACTATCC	GTTCTATCCGAGCAACTGGC
MF347306.1	GTGGTGGTGGTCTAACTATCC	GTTCTATCCGAGCAACTGGC
MF347307.1	GTGGTGGTGGTCTAACTATCC	GTTCTATCCGAGCAACTGGC
MF347312.1	GTGGTGGTGGTCTAACTATCC	GTTCTATCCGAGCAACTGGC
MF347313.1	GTGGTGGTGGTCTAACTATCC	GTTCTATCCGAGCAACTGGC
MF347314.1	GTGGTGGTGGTCTAACTATCC	GTTCTATCCGAGCAACTGGC
MF347323.1	GTGGTGGTGGTCTAACTATCC	GTTCTATCCGAGCAACTGGC
MF347362.1	GTGGTGGTGGTCTAACTATCC	GTTCTATCCGAGCAACTGGC

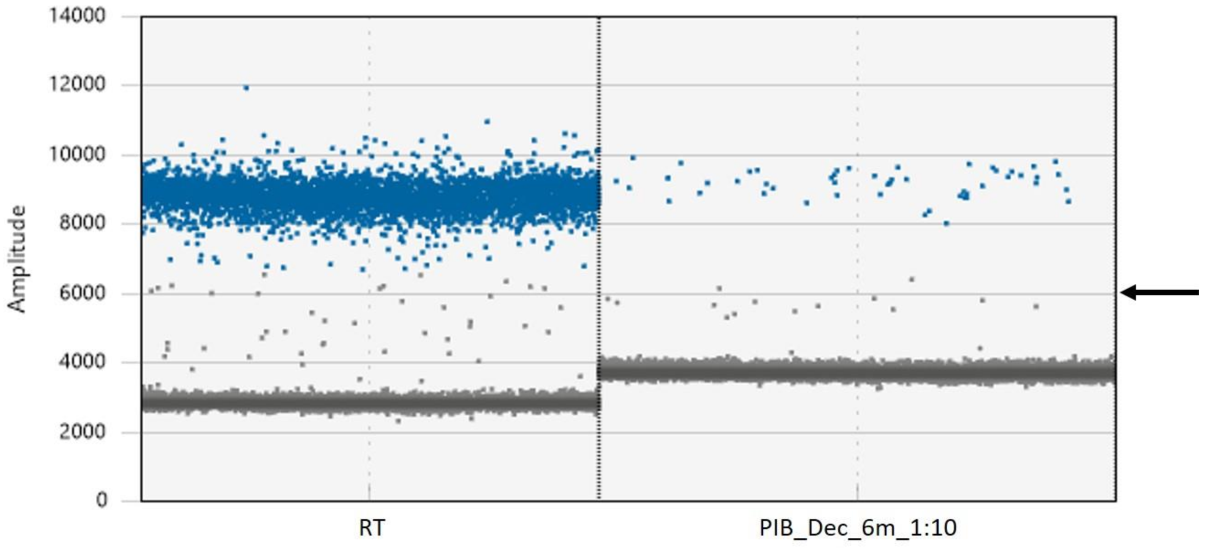

Figure S10: Fluorescence amplitudes of ddPCR droplets produced with ComaA1A2_336F/497R primer pair (primer details are given in Table 1) in samples of the rainwater tank (left) and pelagic water samples of Piburger See (right). The arrow on the right side shows the fluorescence amplitude of potential primer dimers.

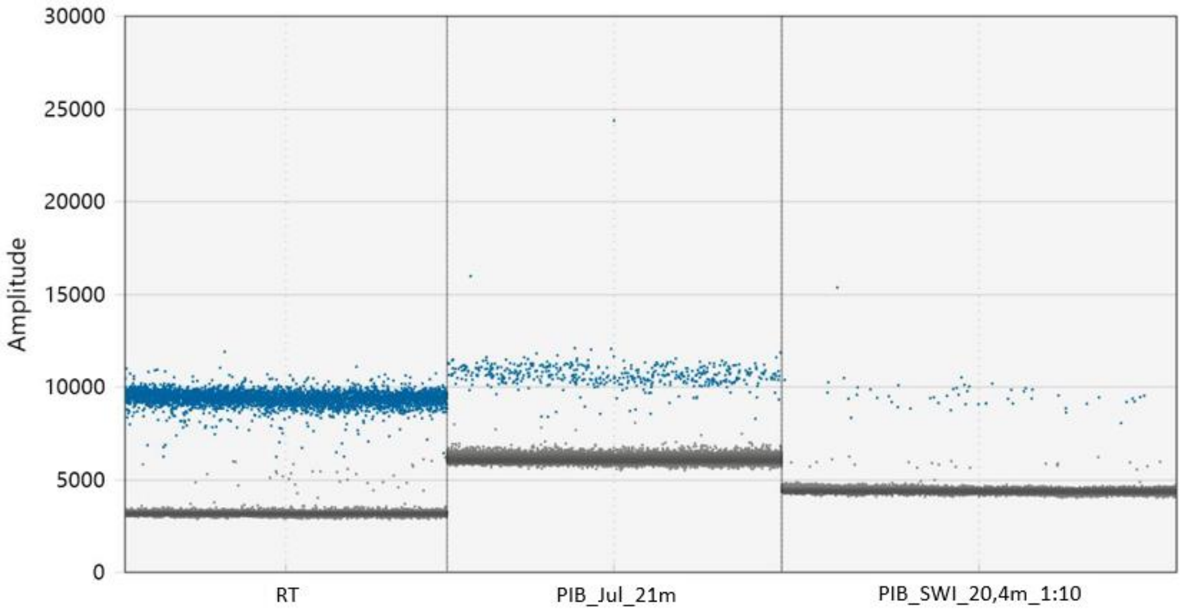

Figure S11: Fuorescence amplitudes of ddPCR droplets produced with ComaA1A2_336F/497R primer pair (primer details are given in Table 1) in samples of the rainwater tank (left), pelagic water samples of Piburger See (middle) and samples of the sediment/water-interface (right).

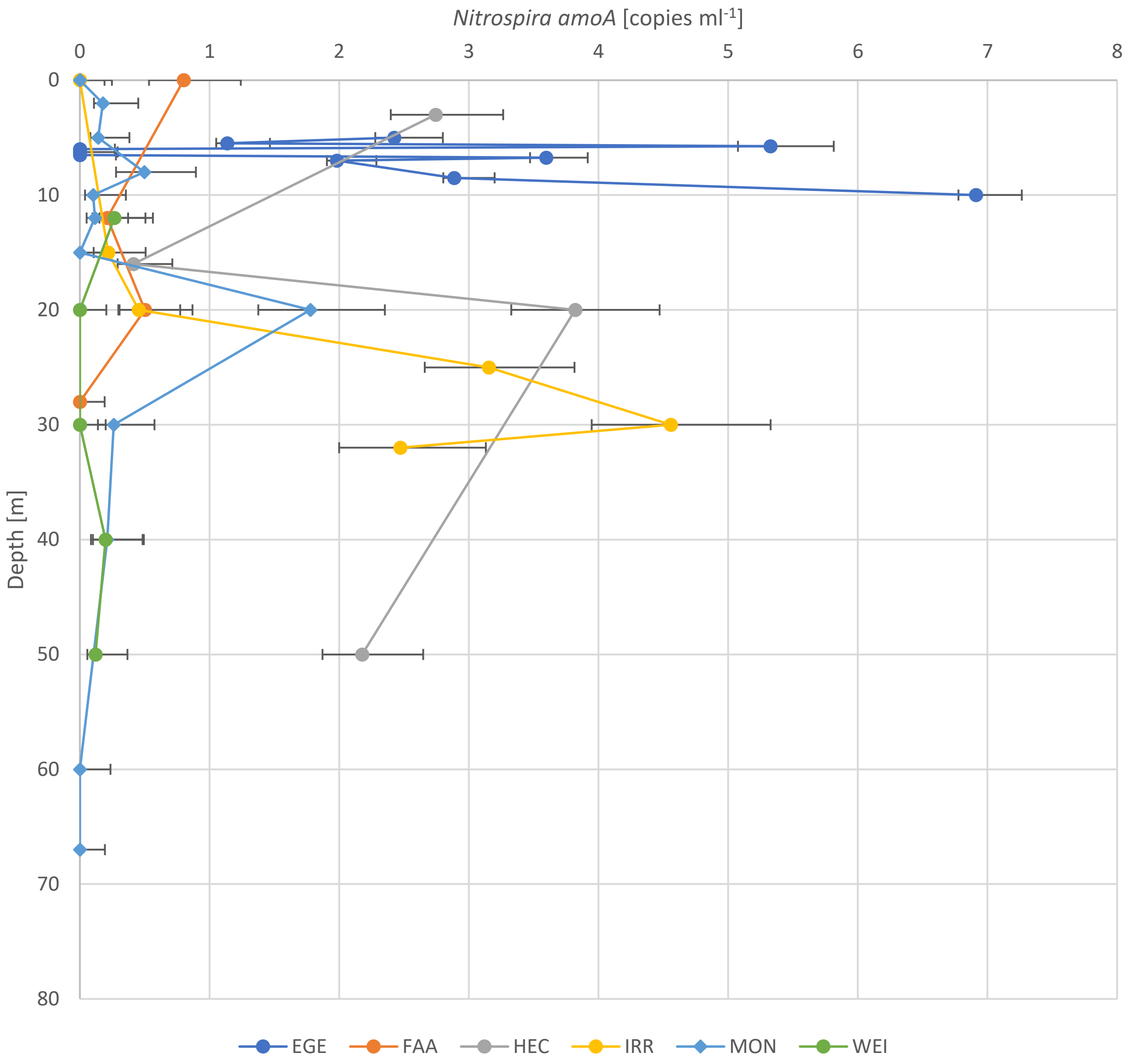

Figure S12: Vertical distribution of *Nitrospira amoA* copies ml^{-1} in lakes with a depth <100 m. Error bars indicate 95% poisson confidence intervals. EGE= Egelsee, FAA= Faakersee, HEC= Hechtsee, IRR= Irrsee, MON= Mondsee, WEI= Weißensee.

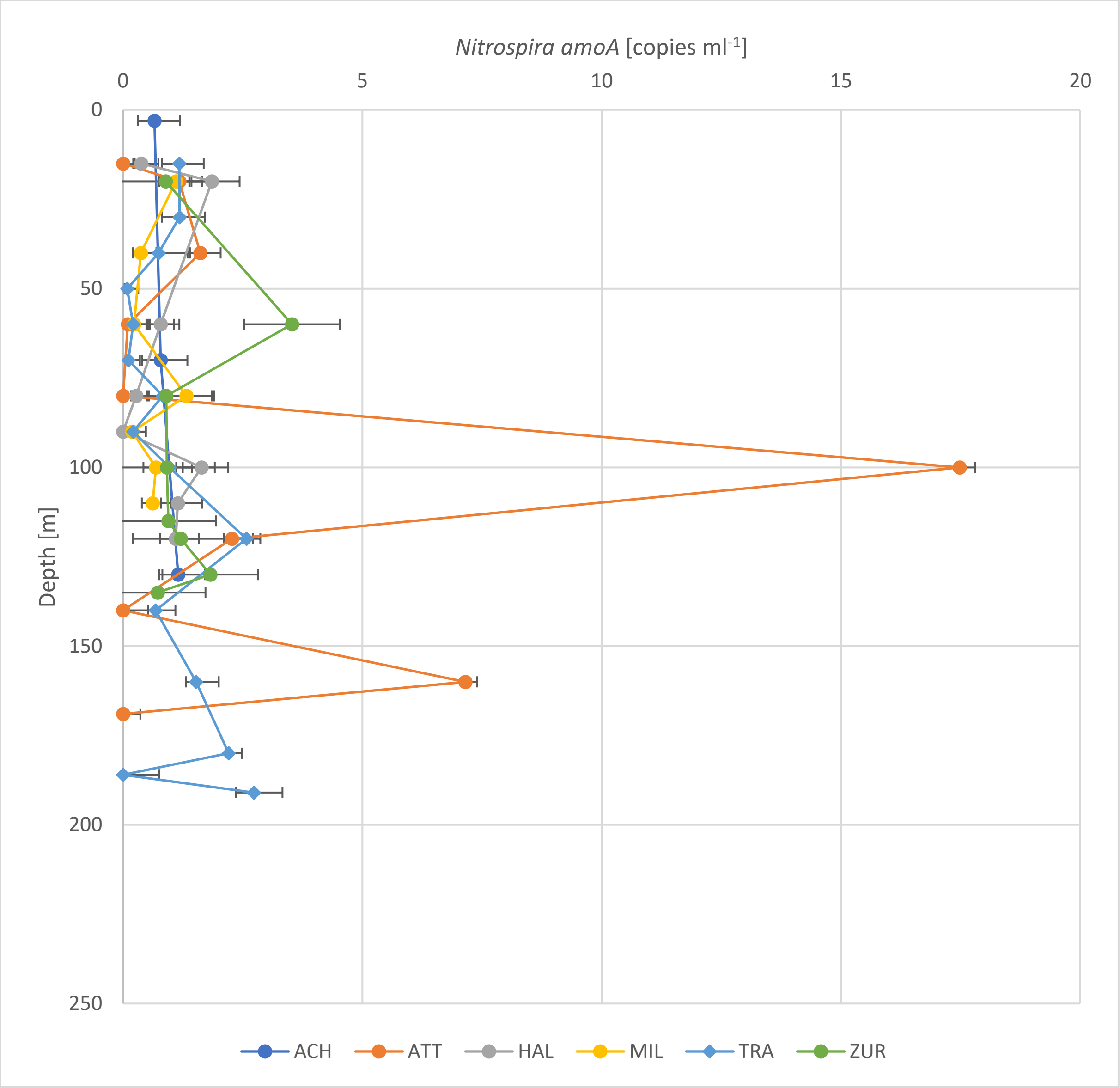

Figure S13: Vertical distribution of *Nitrospira amoA* copies ml^{-1} in lakes with a depth >100 m. Error bars indicate 95% poisson confidence intervals. ACH= Achensee, ATT= Attersee, HAL= Hallstättersee, MIL= Millstätter See, TRA= Traunsee, ZUR= Zürichsee.
